# An algorithm for simplified hepatitis C virus treatment with non-specialist care based on nation-wide data from Taiwan

**DOI:** 10.1007/s12072-023-10609-7

**Published:** 2024-01-21

**Authors:** Ming-Lung Yu, Chi‐Ming Tai, Lein-Ray Mo, Hsing-Tao Kuo, Chung-Feng Huang, Kuo-Chih Tseng, Ching-Chu Lo, Ming-Jong Bair, Szu-Jen Wang, Jee-Fu Huang, Ming-Lun Yeh, Chun-Ting Chen, Ming-Chang Tsai, Chien-Wei Huang, Pei-Lun Lee, Tzeng-Hue Yang, Yi-Hsiang Huang, Lee-Won Chong, Chien-Lin Chen, Chi-Chieh Yang, Chao-Hung Hung, Sheng‐Shun Yang, Pin-Nan Cheng, Tsai-Yuan Hsieh, Jui-Ting Hu, Wen-Chih Wu, Chien-Yu Cheng, Guei-Ying Chen, Guo-Xiong Zhou, Wei-Lun Tsai, Chien-Neng Kao, Chih-Lang Lin, Chia-Chi Wang, Ta-Ya Lin, Chih‐Lin Lin, Wei-Wen Su, Tzong-Hsi Lee, Te-Sheng Chang, Chun-Jen Liu, Chia-Yen Dai, Chi-Yi Chen, Jia-Horng Kao, Han-Chieh Lin, Wan-Long Chuang, Cheng-Yuan Peng

**Affiliations:** 1 School of Medicine, College of Medicine and Center of Excellence for Metabolic Associated Fatty Liver Disease, National Sun Yat-Sen University, No. 70, Lianhai Rd, Gushan District, Kaohsiung City, Taiwan 804; 2 Hepatobiliary Division, Department of Internal Medicine and Hepatitis Center, Kaohsiung Medical University Hospital, Kaohsiung Medical University, No. 100, Shihcyuan 1st Rd, Sanmin District, Kaohsiung City, Taiwan 807; 3 Hepatitis Research Center, College of Medicine and Center for Liquid Biopsy and Cohort Research, Kaohsiung Medical University, No. 100, Shihcyuan 1st Rd, Sanmin District, Kaohsiung City, Taiwan 807; 4 Division of Hepatogastroenterology, Department of Internal Medicine, Kaohsiung Chang Gung Memorial Hospital, No. 123, Dapi Rd, Niaosong District, Kaohsiung City, Taiwan 833; 5 Division of Gastroenterology and Hepatology, Department of Internal Medicine, E-Da Hospital, I-Shou University, No. 1, Section 1, Xuecheng Rd, Dashu District, Kaohsiung City, Taiwan 840; 6 School of Medicine for International Students, College of Medicine, I-Shou University, No. 1, Section 1, Xuecheng Rd, Dashu District, Kaohsiung City, Taiwan 840; 7 Division of Gastroenterology, Tainan Municipal Hospital (Managed By Show Chwan Medical Care Corporation), No. 670, Chongde Rd, East District, Tainan City, Taiwan 701; 8 Division of Gastroenterology and Hepatology, Department of Internal Medicine, Chi Mei Medical Center, No. 901, Zhonghua Rd, Yongkang District, Tainan City, Taiwan 710; 9 School of Medicine, College of Medicine, National Sun Yat-Sen University, No. 70, Lianhai Rd, Gushan District, Kaohsiung City, Taiwan 804; 10 Ph.D. Program in Translational Medicine, College of Medicine, Kaohsiung Medical University and Academia Sinica, No. 128, Section 2, Academia Rd, Nangang District, Taipei City, Taiwan 115; 11 Department of Internal Medicine, Dalin Tzu Chi Hospital, Buddhist Tzu Chi Medical Foundation, No. 2, Minsheng Rd, Dalin Township, Chiayi County, Taiwan 622; 12 School of Medicine, Tzuchi University, No. 701, Section 3, Zhongyang Rd, Hualien City, Hualien County, Taiwan 970; 13 Division of Gastroenterology, Department of Internal Medicine, St. Martin De Porres Hospital, No. 60, Minquan Rd, East District, Chiayi City, Taiwan 600; 14 Division of Gastroenterology, Department of Internal Medicine, Taitung Mackay Memorial Hospital, No. 1, Lane 303, Zhangsha St, Taitung City, Taitung County Taiwan 950; 15 Mackay Medical College, No. 46, Section 3, Zhongzheng Rd, Sanzhi District, New Taipei City, Taiwan 252; 16 Division of Gastroenterology, Department of Internal Medicine, Yuan’s General Hospital, No. 162, Chenggong 1st Rd, Lingya District, Kaohsiung City, Taiwan 802; 17 Division of Gastroenterology, Department of Internal Medicine, Tri-Service General Hospital, National Defense Medical Center, No. 325, Section 2, Chenggong Rd, Neihu District, Taipei City, Taiwan 114; 18 Division of Gastroenterology, Department of Internal Medicine, Tri-Service General Hospital Penghu Branch, National Defense Medical Center, No. 90, Qianliao, Magong City, Penghu County Taiwan 880; 19 School of Medicine, Chung Shan Medical University, Department of Internal Medicine, Chung Shan Medical University Hospital, No. 110, Section 1, Jianguo N Rd, South District, Taichung City, Taiwan 402; 20 Division of Gastroenterology, Kaohsiung Armed Forces General Hospital, No. 2, Zhongzheng 1st Rd, Lingya District, Kaohsiung City, Taiwan 802; 21 Lotung Poh-Ai Hospital, No. 83, Nanchang St, Luodong Township, Yilan County Taiwan 265; 22 Division of Gastroenterology and Hepatology, Department of Medicine, Taipei Veterans General Hospital, No. 201, Section 2, Shipai Rd, Beitou District, Taipei City, Taiwan 112; 23 Institute of Clinical Medicine, School of Medicine, National Yang-Ming Chiao Tung University, No. 155, Section 2, Linong St, Beitou District, Taipei City, Taiwan 112; 24 Division of Hepatology and Gastroenterology, Department of Internal Medicine, Shin Kong Wu Ho-Su Memorial Hospital, Shilin District, Taipei City, Taiwan 111; 25 School of Medicine, Fu-Jen Catholic University, No. 510, Zhongzheng Rd, Xinzhuang District, New Taipei City, Taiwan 242; 26 Department of Medicine, Hualien Tzu Chi Hospital, Buddhist Tzu Chi Medical Foundation and Tzu Chi University, No. 701, Section 3, Zhongyang Rd, Hualien City, Hualien County Taiwan 970; 27 Department of Gastroenterology, Division of Internal Medicine, Show Chwan Memorial Hospital, No. 542, Section 1, Zhongshan Rd, Changhua City, Changhua County Taiwan 500; 28 Division of Gastroenterology and Hepatology, Department of Internal Medicine, Taichung Veterans General Hospital, No. 1650, Section 4, Taiwan Boulevard, Xitun District, Taichung City, Taiwan 407; 29 Division of Gastroenterology and Hepatology, Department of Internal Medicine, National Cheng Kung University Hospital, College of Medicine, National Cheng Kung University, No. 1, Dasyue Rd, East District, Tainan City, Taiwan 701; 30 Liver Center, Cathay General Hospital, No. 280, Section 4, Ren’ai Rd, Da’an District, Taipei City, Taiwan 106; 31 Wen-Chih Wu Clinic, Fengshan, Kaohsiung, Taiwan 830; 32 Division of Infectious Diseases, Department of Internal Medicine, Taoyuan General Hospital, Ministry of Health and Welfare, No. 1492, Zhongshan Rd, Taoyuan District, Taoyuan City, Taiwan 330; 33 Institute of Public Health, School of Medicine, National Yang-Ming Chiao Tung University, No. 155, Section 2, Linong St, Beitou District, Taipei City, Taiwan 112; 34 Penghu Hospital, Ministry of Health and Welfare, No. 10, Zhongzheng Rd, Magong City, Penghu County Taiwan 880; 35 Zhou Guoxiong Clinic, Penghu, Taiwan 880; 36 Kaohsiung Veterans General Hospital, No. 386, Dazhong 1st Rd, Zuoying District, Kaohsiung City, Taiwan 813; 37 National Taiwan University Hospital Hsin-Chu Branch, No. 25, Lane 442, Section 1, Jingguo Rd, North District, Hsinchu City, Taiwan 300; 38 Liver Research Unit, Department of Hepato-Gastroenterology and Community Medicine Research Center, Chang Gung Memorial Hospital at Keelung, College of Medicine, Chang Gung University, No. 222, Maijin Rd, Anle District, Keelung City, Taiwan 204; 39 Taipei Tzu Chi Hospital, Buddhist Tzu Chi Medical Foundation and School of Medicine, Tzu Chi University, No. 289, Jianguo Rd, Xindian District, New Taipei City, Taiwan 231; 40 Cishan Hospital, Ministry of Health and Welfare, No. 60, Zhongxue Rd, Qishan District, Kaohsiung City, Taiwan 842; 41 Department of Gastroenterology, Renai Branch, Taipei City Hospital, No. 10, Section 4, Ren’ai Rd, Da’an District, Taipei City, Taiwan 106; 42 Department of Gastroenterology and Hepatology, Changhua Christian Hospital, No. 176, Zhonghua Rd, Changhua City, Changhua County, Taiwan 500; 43 Division of Gastroenterology and Hepatology, Far Eastern Memorial Hospital, No. 21, Section 2, Nanya S Rd, Banqiao District, New Taipei City, Taiwan 220; 44 Division of Hepatogastroenterology, Department of Internal Medicine, Chang Gung Memorial Hospital, Chiayi, Taiwan and College of Medicine, Chang Gung University, No. 259, Wenhua 1st Rd, Guishan District, Taoyuan City, Taiwan 333; 45 Hepatitis Research Center and Department of Internal Medicine, National Taiwan University Hospital, No. 1, Section 4, Roosevelt Rd, Da’an District, Taipei City, Taiwan 106; 46 Division of Gastroenterology and Hepatology, Department of Medicine, Ditmanson Medical Foundation Chiayi Christian Hospital, No. 539, Zhongxiao Rd, East District, Chiayi City, Taiwan 600; 47 Center for Digestive Medicine, Department of Internal Medicine, China Medical University Hospital, No. 2, Yude Rd, North District, Taichung City, Taiwan 404; 48 School of Medicine, China Medical University, No. 91, Xueshi Rd, North District, Taichung City, Taiwan 404

**Keywords:** Simplified treatment, Direct-acting antivirals, Safety, Laboratory abnormalities, Liver function, Taiwan hepatitis C registry, European Association for the Study of the Liver, American Association for the Study of Liver Diseases and the Infectious Diseases Society of America, sofosbuvir/velpatasvir, glecaprevir/pibrentasvir

## Abstract

**Background:**

Both European Association for the Study of the Liver (EASL) and American Association for the Study of Liver Diseases and the Infectious Diseases Society of America (AASLD-IDSA) guidelines recommend simplified hepatitis C virus (HCV) treatment with pan-genotypic sofosbuvir/velpatasvir or glecaprevir/pibrentasvir for eligible patients. This observational study used real-world data to assess these regimens’ safety in eligible patients and develop an algorithm to identify patients suitable for simplified treatment by non-specialists.

**Methods:**

7,677 HCV-infected patients from Taiwan Hepatitis C Registry (TACR) who received at least one dose of sofosbuvir/velpatasvir or glecaprevir/pibrentasvir, and fulfilled the EASL/AASLD-IDSA criteria for simplified treatment were analyzed. Multivariate analysis was conducted on patient characteristics and safety data.

**Results:**

Overall, 92.8% (7,128/7,677) of patients achieved sustained virological response and only 1.9% (146/7,677) experienced Grades 2–4 laboratory abnormalities in key liver function parameters (alanine aminotransferase, aspartate aminotransferase, and total bilirubin), with only 18 patients (0.23%) experiencing Grades 3–4 abnormalities. Age > 70 years old, presence of hepatocellular carcinoma, total bilirubin > 1.2 mg/dL, estimated glomerular filtration rate < 60 mL/min/1.73 m^2^, and Fibrosis-4 > 3.25 were associated with higher risks of Grades 2–4 abnormalities. Patients with any of these had an odds of 4.53 times than that of those without in developing Grades 2–4 abnormalities (*p* < 0.01).

**Conclusions:**

Real-world data from Taiwan confirmed that simplified HCV treatment for eligible patients with pan-genotypic regimens is effective and well tolerated. The TACR algorithm, developed based on this study’s results, can further identify patients who can be safely managed by non-specialist care.

**Graphical Abstract:**

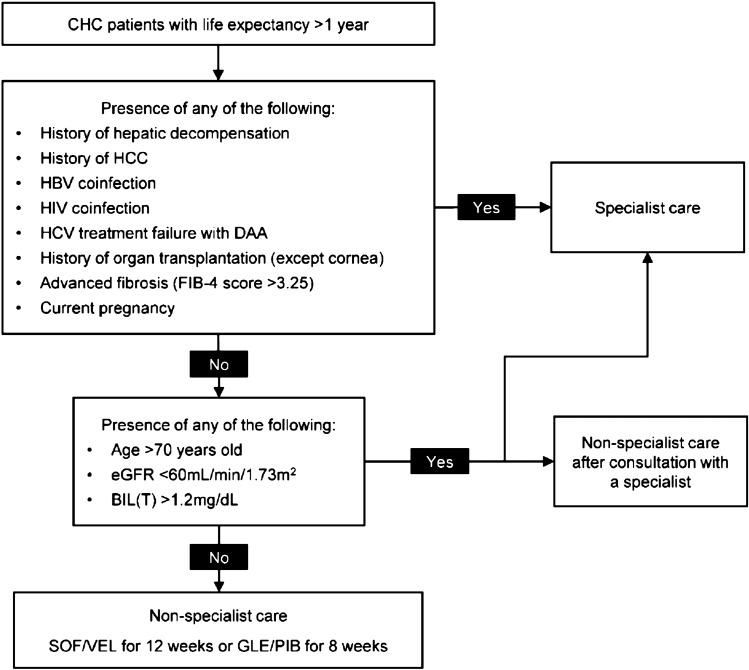

**Supplementary Information:**

The online version contains supplementary material available at 10.1007/s12072-023-10609-7.

## Introduction

The development of highly effective, well-tolerated, pan-genotypic direct-acting antiviral agents (DAAs) has greatly simplified the treatment of HCV infection; however, limited access to HCV care remains an obstacle to HCV elimination [1]. A recent modeling study showed that among 56.8 million people infected with HCV in 2020, an estimated 23% was diagnosed, and only 5% of those diagnosed were initiated on treatment [2]. This is partly due to a lack of specialist resources (such as hepatologists, gastroenterologists, infectious disease specialists) to manage HCV infection [1]. As highlighted by the World Health Organization (WHO), decentralized service delivery and task-shifting to non-specialists are crucial for expanding the access to HCV treatment [3], and several systematic reviews have demonstrated that non-specialist care (e.g., by general practitioners, family doctors, and nurses) can achieve similar sustained virological response (SVR) rates with DAA treatment compared with those obtained by specialists [4, 5].

Simplified HCV treatment algorithms based on standard blood and fibrosis tests are essential for decentralized service delivery [1, 6]. Besides reducing pre-treatment evaluation and on-treatment monitoring, simplified treatment algorithms can facilitate task-sharing by directing non-complicated HCV infection cases to non-specialist care and directing patients with more complex disease status (e.g., with decompensated cirrhosis) to appropriate specialist care [1, 4, 6–8]. For this purpose, suitable patient eligibility criteria must be in place to identify patients who can be safely managed by non-specialists: while unduly stringent criteria might hamper care decentralization, lenient criteria may cause some complicated cases to be assigned to non-specialist care, putting these patients at higher risks of safety complications, such as liver function abnormalities.

Both the American Association for the Study of Liver Diseases and the Infectious Diseases Society of America (AASLD-IDSA) and the European Association for the Study the Liver (EASL) have recommended simplified, genotyping/subtyping-free treatment with sofosbuvir/velpatasvir (SOF/VEL) or glecaprevir/pibrentasvir (GLE/PIB), with clear eligibility criteria for simplified treatment [8, 9]. These guidelines-recommended algorithms are intended for specialists [1]; their eligibility criteria were formulated based on expert opinion and are not entirely consistent (possibly due to potential population differences or different needs of regional HCV care infrastructure) [8, 9]. Although SOF/VEL and GLE/PIB have well-established efficacy and are generally well tolerated [6, 8, 10], they are not completely free from adverse events [11, 12]. Currently, the real-world safety of implementing the AASLD-IDSA and the EASL simplified treatment algorithms has not been extensively studied, and real-world data are lacking on how effectively their eligibility criteria can distinguish between patient populations with less and more safety management needs. Therefore, the existing sets of eligibility criteria may be inadequate when simplified treatment is extended into the non-specialist setting.

In Taiwan, the estimated prevalence of HCV infection is 3.28% in the general population and > 10% in some hyperendemic areas [13]. Following WHO’s recommendation for decentralization and task-sharing, Taiwan has relaxed the local treatment guidance to allow all physicians, including non-specialists, to prescribe DAAs for HCV treatment since October 2021 [14]. As such, there is an urgent need for a set of validated criteria to identify non-complicated chronic hepatitis C (CHC) patients suitable for simplified treatment with non-specialist care. Other countries that have rolled out non-specialist HCV care, such as Australia [15], may have similar needs for guidelines and standards.

The Taiwan Hepatitis C Registry Program (TACR), established by the Taiwan Association for the Study of the Liver (TASL), is a nation-wide registry managing the database and biobank of DAA-treated CHC patients across numerous participating centers in Taiwan and contains well-documented baseline and regular on-treatment laboratory monitoring data [16]. As of September 1st, 2022, TACR included 53 participating sites and 41,253 CHC patients, accounting for one-third of the DAA-treated patients in Taiwan. We utilized the real-world safety data of SOF/VEL- or GLE/PIB-treated patients in TACR to validate the AASLD-IDSA and EASL criteria for simplified treatment, and to develop an algorithm (“the TACR algorithm”) to identify CHC patients eligible for simplified treatment with non-specialist care.

## Materials and methods

### Patients

In this retrospective prospective analysis, CHC patients registered in TACR from August 2019 to August 2021 were screened, and were included if they received at least one dose of SOF/VEL or GLE/PIB and fulfilled the criteria for simplified treatment by either AASLD-IDSA (treatment-naïve adult CHC patients without cirrhosis or with compensated cirrhosis, and without any of the following conditions: human immunodeficiency virus (HIV) or HBV coinfection, current pregnancy, history of hepatocellular carcinoma (HCC) or prior liver transplantation, compensated cirrhosis with stage 4 or 5 chronic kidney disease [CKD]) or EASL (HCV mono-infected or HCV–HIV-coinfected adults and adolescences, DAA-naïve except for prior SOF/pegylated interferon/ribavirin treatment, without cirrhosis or with compensated cirrhosis, and without HBV coinfection or current pregnancy) [8, 9]. Patients were excluded if they had missing aspartate aminotransferase (AST), alanine aminotransferase (ALT), or total bilirubin (BIL[T]) data at baseline or the end of the treatment, or if they discontinued the study treatment prematurely and had no AST, ALT, or BIL(T) data after baseline. SOF/VEL and GLE/PIB treatment conformed to the regional consensus recommendations or the regulations of the National Health Insurance Administration of Taiwan [17–19]. Briefly, patients received 12 weeks of SOF/VEL treatment or 8–12 weeks of GLE/PIB treatment, and were followed for a minimum of three months after completing the treatment. This study was approved by the institutional review board at each study site and conformed to the guidelines of the International Conference on Harmonization for Good Clinical Practice. All patients provided written informed consent.

### Endpoints

Patient demographic and baseline characteristics were collected, including age, sex, HCV viral load and genotype, liver cirrhosis status, renal function, comorbidities, history of previous HCV treatment, HCC, liver transplantation, and drug abuse. Definition of liver cirrhosis has been previously described [20]. Patients with CKD included dialytic patients and patients with an estimated glomerular filtration rate (eGFR, by the Modification of Diet in Renal Disease [MDRD] equation [21]) < 60 mL/min/1.73 m^2^ or evidence of kidney function damage (e.g., presence of proteinuria) for more than three months.

The primary endpoints were the proportions of patients with Grades 2–4 laboratory abnormalities in ALT, AST, and BIL(T) during treatment and the three-month posttreatment follow-up period. These parameters were chosen because SOF/VEL and GLE/PIB are generally well tolerated, with low incidences of adverse events (AEs) and few AEs leading to treatment discontinuation [7, 11, 22], while abnormal elevations in liver function parameters could lead to treatment discontinuation, resulting in the need for specialist care [8]. Abnormal elevations in AST, ALT, and BIL(T) were defined according to Common Terminology Criteria for Adverse Events v5.0 [23]: for AST/ALT levels, if baseline (BL) was normal (≤ 40 U/L), Grades 2, 3, and 4 abnormal elevations were defined as ≥ 3.0–5.0 × upper limit of normal (ULN), > 5.0–20.0 × ULN, and > 20.0 × ULN, respectively; if BL was elevated (> 1 × ULN), Grades 2, 3, and 4 abnormal elevations were defined as > 3.0–5.0 × BL, > 5.0–20.0 × BL, and > 20.0 × BL, respectively. Grades 2, 3, and 4 abnormal elevations in BIL(T) were defined as > 1.5–3.0 × ULN, > 3.0–10.0 × ULN, and > 10 × ULN, respectively, for patients with normal BL (≤ 1.2 mg/dL), and as > 1.5–3.0 × BL, > 3.0–10.0 × BL, and > 10 × BL, respectively, for patients with elevated BL (> 1 × ULN).

### Statistical analyses

Baseline demographic and clinical characteristics were summarized using descriptive statistics (mean ± SD and patient number [percentage]). Frequencies were compared between groups using the *χ*^2^ test with the Yates correction or the Fisher’s exact test. Group means were compared using analysis of variance, Student’s t-test, or the nonparametric Mann–Whitney *U* test when appropriate. Multivariate logistic regression analysis was performed to determine factors associated with the occurrence of laboratory abnormalities in liver function parameters by analyzing the covariates with *p* < 0.10 in the univariate analysis. To compare the ability of the different models for simplification in predicting the occurrence of abnormal elevations in ALT/AST/BIL(T), the goodness of fit was assessed by Akaike information criterion (AIC) and Schwartz’s Bayesian information criterion (BIC). Both were based on the maximum likelihood estimates of the model parameters, and a smaller value was considered an indicator of better fit. The formulae were as follows: AIC = −2 ln(L) + 2 k; BIC = −2 ln(L) + ln(n) × k, where k was the number of parameters and L was the likelihood function. Two-sided hypothesis tests with a significance level of *p* < 0.05 were used for all statistical analyses. All statistical analyses were performed using the SPSS 12.0 statistical package (SPSS, Chicago, IL, USA).

## Results

### Patient characteristics

**Fig. 1:**
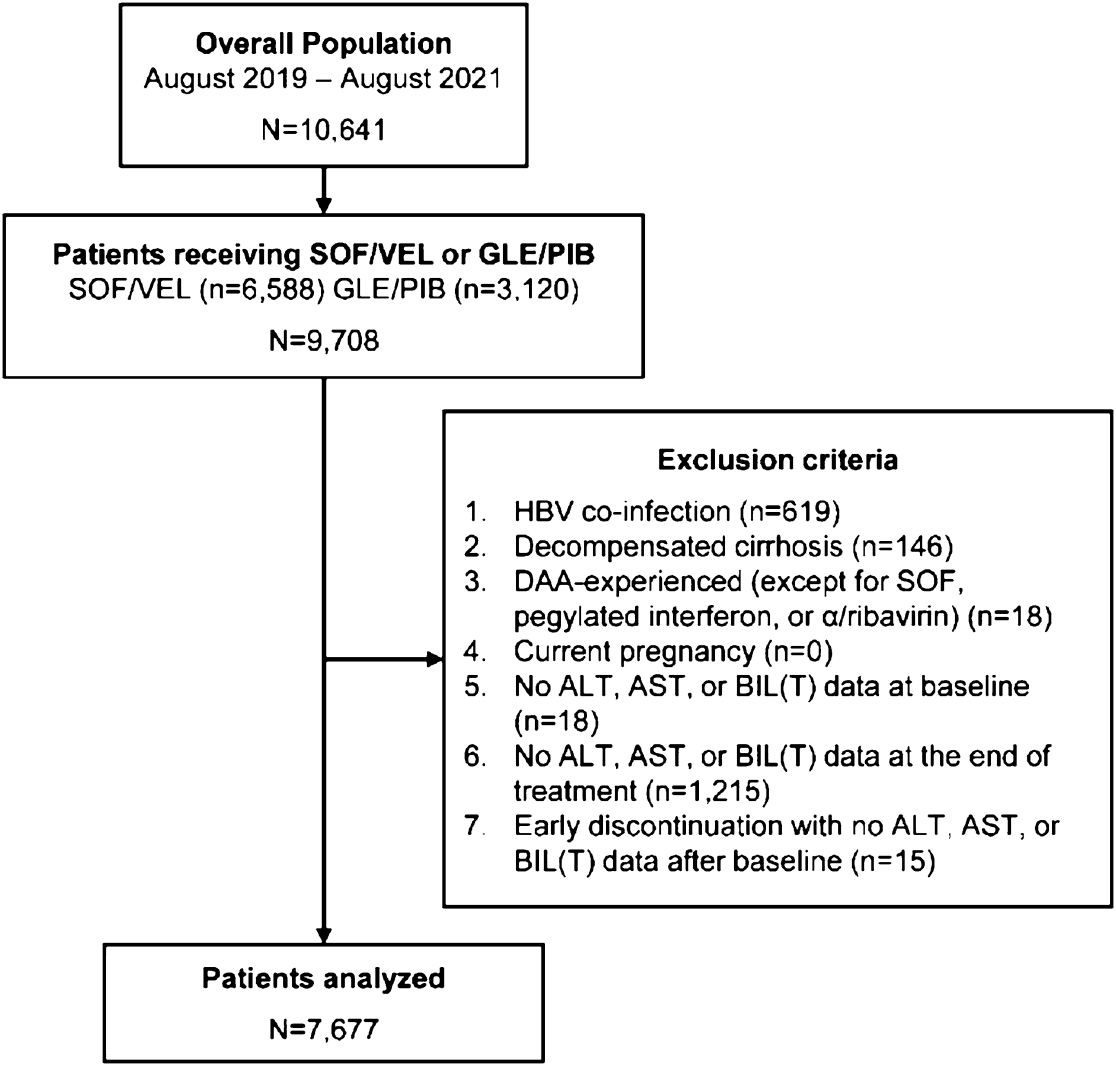
Patient flow chart. ALT, alanine aminotransferase, AST, aspartate aminotransferase, BIL(T), total bilirubin, DAA, direct-acting agents, GLE/PIB, glecaprevir/pibrentasvir, SOF: sofosbuvir, SOF/VEL, sofosbuvir/velpatasvir

From August 2019 to August 2021, 10,641 patients were registered in TACR and 9,708 received SOF/VEL or GLE/PIB treatment (Fig. 1). Among them, 7,677 met the inclusion criteria and were analyzed. Baseline characteristics are summarized in Table 1. The mean age was 59.6 years old, and 53.0% of the patients were male. The most common HCV genotypes were genotype 2 (44.0%) and 1 (38.0%). Among all, 1,546 (20.2%) patients had baseline HCV RNA > 6,000,000 IU/mL, 798 (10.0%) patients had compensated liver cirrhosis, and 283 (3.7%) patients had HCC. Most patients (97.8%) were treatment naïve at baseline. SOF/VEL and GLE/PIB were used to treat 68.1% and 31.9% of the patients, respectively.

**Table 1:** Patient baseline characteristics

Characteristics	Total (*n* = 7,677)
Age (years), mean ± SD	59.6 ± 13.6
Male, *n* (%)	4,066 (53.0)
BMI (kg/m^2^), mean ± SD	24.5 ± 4.1
AST (U/L), mean ± SD	53.9 ± 55.6
ALT (U/L), mean ± SD	67.5 ± 85.0
Creatinine (mg/dL), mean ± SD	1.1 ± 1.4
BIL(T) (mg/dL), mean ± SD	0.8 ± 0.4
Platelet count, ×10^3^u/L	202.0 ± 72.4
FIB-4, mean ± SD > 3.25, *n* (%)^a^	2.5 ± 2.5 1,650 (21.6)
eGFR, mL/min/1.73 m^2^, mean ± SD	88.1 ± 31.4
Treatment history	
Naïve, *n* (%)	7,509 (97.8)
Experienced, *n* (%)	168 (2.2)
IFN/DAA^b^, *n* (%)	163 (2.1)/5 (0.1)
Diabetes, *n* (%)	1,245 (16.2)
Hypertension, *n* (%)	2,246 (29.3)
Hyperlipidemia, *n* (%)	851 (11.1)
Cardiovascular disease, *n* (%)	644 (8.4)
HIV coinfection, *n* (%)	210 (2.7)
PWID, *n* (%)	818 (10.7)
Compensated liver cirrhosis, *n* (%)^c^	798 (10.0)
HCC, *n* (%)	283 (3.7)
Liver transplantation, *n* (%)	5 (0.1)
HCV RNA log_10_IU/mL, mean ± SD > 600 KIU/mL, *n* (%)^d^	5.9 ± 1.1 1,546 (20.2)
HCV genotype, *n* (%)	
1	2,915 (38.0)
2	3,376 (44.0)
3	227 (3.0)
4	2 (0.03)
5	1 (0.01)
6	933 (12.2)
Mixed	137 (1.8)
Unclassified	86 (1.1)
Regimen, *n* (%)	
SOF/VEL	5,228 (68.1)
GLE/PIB	2,449 (31.9)

^a^*n* = 7,658, 19 patients had no available FIB-4 data

^b^DAA experience: SOF/RBV

^c^*n* = 7,664 (13 patients had no available liver cirrhosis data)

^d^*n* = 7,670 (7 patients had no available HCV RNA data)

*ALT* alanine aminotransferase, *AST* aspartate aminotransferase, *BMI* body mass index, *BIL(T)* total bilirubin, *DAA* direct-acting agents, *eGFR* estimated glomerular filtration rate by the Modification of Diet in Renal Disease equation, *FIB-4* fibrosis-4, *GLE/PIB* glecaprevir/pibrentasvir, *HCC* hepatocellular carcinoma, *HIV* human immunodeficiency virus, *IFN* interferon, *PWID* persons who inject drugs, *SOF/VEL* sofosbuvir/velpatasvir

### Overall efficacy and safety

Overall, 7,128 of the 7,677 patients (92.8%) achieved SVR at posttreatment week 12 (SVR12), 60 (0.8%) patients did not achieve SVR12, and the remaining 489 (6.4%) patients had unknown virologic outcomes, giving rise to an SVR12 rate of 99.1% (7,128/7,188) by per protocol analysis. A total of 146 patients (1.9%) experienced Grades 2–4 laboratory abnormalities in ALT/AST/BIL(T) during treatment and the three-month posttreatment follow-up period. The incidences of abnormal elevations in ALT/AST/BIL(T) were low and were mostly Grade 2 BIL(T) elevation (1.5%). Very few patients (0.23%) had Grades 3–4 abnormal elevations in these parameters (Table 2). Characteristics of patients who experienced Grades 3–4 abnormal elevations in ALT/AST/BIL(T) are summarized in Supplementary Table 1.

**Table 2:** Frequency and distribution of laboratory abnormalities in liver function parameters

*n* (%)	Grade 2	Grade 3	Grade 4
BIL(T)	116 (1.5)	6 (0.1)	0 (0.0)
AST	7 (0.1)	8 (0.1)	0 (0.0)
ALT	12 (0.2)	8 (0.1)	1 (0.01)
ALT/AST/BIL(T)	**128 (1**.**6)**	**17 (0**.**2)**	**1 (0**.**01)**

*ALT* alanine aminotransferase, *AST* aspartate aminotransferase, *BIL(T)* total bilirubin

### Risk factors associated with abnormal elevations in liver function parameters

Univariate and subsequent multivariate logistic regression analyses identified six factors associated with the occurrence of Grades 2–4 abnormal elevations in ALT/AST/BIL(T), namely age > 70, presence of HCC, BIL(T) > 1.2 mg/dL, eGFR < 60 mL/min/1.73 m^2^, FIB-4 > 3.25, and GLE/PIB usage (versus SOF/VEL) (Table 3). Separate risk factor analyses for each of the three liver function parameters (Grades 2–4) can be found in Supplementary Tables 2–4, and that among patients treated with SOF/VEL or GLE/PIB can be found in Supplementary Tables 5 and 6. For Grades 3–4 laboratory abnormalities, the factors of age > 70, FIB-4 > 3.25, and GLE/PIB usage emerged in univariate analyses while only GLE/PIB usage remained significant in multivariate analyses (Table 4). It was mainly due to a difference in BIL(T) elevation (GLE/PIB, 0.2% [*n* = 6] vs. SOF/VEL, 0% [*n* = 0], *p* = 0.001), not ALT and/or AST elevation (GLE/PIB, 0.20% [*n* = 5] vs. SOF/VEL, 0.1% [*n* = 7], *p* = 0.468). Analyses focusing on GLE/PIB-treated patients (Supplementary Table 7) identified age > 70, presence of HCC, baseline BIL(T) > 1.2 mg/dL, and FIB-4 > 3.25 as risk factors for Grades 2–4 abnormalities in BIL(T).

**Table 3:** Risk factors associated with Grades 2–4 laboratory abnormalities in ALT/AST/BIL(T)

	Grades 2–4 ALT/AST/BIL(T), n/N (%)	Univariate			Multivariate		
		OR	95% CI	P	OR	95% CI	P
Age							
≤ 70	89/5,966 (1.5)	Ref			Ref		
> 70	57/1,711 (3.3)	2.28	1.62–3.19	< 0.01*	1.63	1.11–2.42	0.01*
Sex							
Female	62/3,611 (1.7)	Ref					
Male	84/4,066 (2.1)	1.21	0.87–1.68	0.26			
Diabetes							
No	116/6,432 (1.8)	Ref					
Yes	30/1,245 (2.4)	1.34	0.90–2.01	0.15			
Hypertension							
No	82/5,431 (1.5)	Ref			Ref		
Yes	64/2,246 (2.9)	1.91	1.37–2.66	< 0.01*	1.30	0.89–1.90	0.17
Hyperlipidemia							
No	127/6,826 (1.9)	Ref					
Yes	19/851 (2.2)	1.20	0.74–1.96	0.45			
CVD							
No	123/7,033 (1.8)	Ref			Ref		
Yes	23/644 (3.6)	2.08	1.32–3.27	< 0.01*	1.36	0.82–2.27	0.24
PWID							
No	136/6,859 (2.0)	Ref					
Yes	10/818 (1.2)	0.61	0.32–1.16	0.14			
HIV							
No	141/7,467 (1.9)	Ref					
Yes	5/210 (2.4)	1.27	0.51–3.13	0.61			
HCC							
No	126/7,394 (1.7)	Ref			Ref		
Yes	20/283 (7.1)	4.39	2.69–7.14	< 0.01*	2.63	1.54–4.50	< 0.01*
Baseline BIL(T)							
≤ 1.2	95/6,837 (1.4)	Ref			Ref		
> 1.2	51/840 (6.1)	4.59	3.24–6.50	< 0.01*	4.82	3.30–7.02	< 0.01*
Baseline AST							
≤ 200	141/7,523 (1.9)	Ref					
> 200	5/154 (3.3)	1.76	0.23–1.41	0.22			
Baseline ALT							
≤ 200	139/7,298 (1.9)	Ref					
> 200	7/379 (1.9)	0.97	0.45–2.09	0.94			
FIB-4							
≤ 3.25	82/6,008 (1.4)	Ref			Ref		
> 3.25	64/1,650 (3.9)	2.92	2.09–4.06	< 0.01*	1.83	1.26–2.67	< 0.01*
eGFR							
≥ 60	106/6,573 (1.6)	Ref			Ref		
< 60	40/1,083 (3.7)	2.34	1.62–3.39	< 0.01*	1.58	1.04–2.39	0.03*
Regimen							
SOF/VEL	54/5,228 (1.0)	Ref			Ref		
GLE/PIB	92/2,449 (3.8)	3.74	2.66–5.25	< 0.01*	4.76	3.33–6.80	< 0.01*
Treatment experience							
IFN							
No	144/7,514 (1.9)	Ref					
Yes	2/163 (1.2)	1.57	0.39–6.41	0.53			
DAA							
No	145/7,672 (1.9)	Ref			Ref		
Yes	1/5 (20.0)	12.98	1.44–116.83	0.02*	6.97	0.71–68.10	0.10

^*^p < 0.05

*ALT* alanine aminotransferase, *AST* aspartate aminotransferase, *BIL(T)* total bilirubin, *CI* confidence interval, *CVD* cardiovascular disease, *DAA* direct-acting agents, *eGFR* estimated glomerular filtration rate by the Modification of Diet in Renal Disease equation, *FIB-4* fibrosis-4, *GLE/PIB* glecaprevir/pibrentasvir, *HCC* hepatocellular carcinoma, *HIV* human immunodeficiency virus, *IFN* interferon, *OR* odds ratio, *PWID* persons who inject drugs, *SOF/VEL* sofosbuvir/velpatasvir

**Table 4:** Risk factors associated with Grades 3–4 laboratory abnormalities in ALT/AST/BIL(T)

	Grades 3–4 ALT/AST/BIL(T), n/N (%)	Univariate			Multivariate		
		OR	95% CI	*P*	OR	95% CI	*P*
Age							
≤ 70	10/5,966 (0.2)	Ref			Ref		
> 70	8/1,711 (0.5)	2.80	1.10–7.10	0.03*	2.55	0.94–6.91	0.07
Sex							
Female	12/3,611 (0.3)	Ref					
Male	6/4,066 (0.2)	1.78	0.67–4.74	0.25			
Diabetes							
No	17/6,432 (0.3)	Ref					
Yes	1/1,245 (0.1)	0.30	0.04–2.28	0.25			
Hypertension							
No	14/5,431 (0.3)	Ref					
Yes	4/2,246 (0.2)	0.69	0.23–2.01	0.51			
Hyperlipidemia							
No	17/6,826 (0.3)	Ref					
Yes	1/851 (0.1)	0.47	0.06–3.55	0.46			
CVD							
No	16/7,033 (0.2)	Ref					
Yes	2/644 (0.3)	1.34	0.31–5.96	0.68			
PWID							
No	15/6,859 (0.2)	Ref					
Yes	3/818 (0.4)	1.68	0.49–5.81	0.41			
HIV							
No	17/7,467 (0.2)	Ref					
Yes	1/210 (0.5)	2.10	0.28–15.83	0.47			
HCC							
No	17/7,394 (0.2)	Ref					
Yes	1/283 (0.4)	1.54	0.20–11.60	0.68			
Baseline BIL(T)							
≤ 1.2	14/6,837 (0.2)	Ref					
> 1.2	4/840 (0.5)	2.33	0.77–7.10	0.14			
Baseline AST							
≤ 200	17/7,523 (0.2)	Ref					
> 200	1/154 (0.7)	2.89	0.38–21.82	0.30			
Baseline ALT							
≤ 200	17/7,298 (0.2)	Ref					
> 200	1/379 (0.3)	1.13	0.15–8.54	0.90			
FIB-4							
≤ 3.25	11/6,008 (0.2)	Ref			Ref		
> 3.25	7/1,650 (0.4)	2.32	0.90–6.00	0.08	1.91	0.69–5.27	0.21
eGFR							
≥ 60	14/6,573 (0.2)	Ref					
< 60	4/1,083 (0.4)	1.74	0.57–5.29	0.33			
Regimen							
SOF/VEL	7/5,228 (0.1)	Ref			Ref		
GLE/PIB	11/2,449 (0.5)	3.37	1.30–8.69	0.01*	3.77	1.45–9.78	0.01*
Treatment experience							
IFN							
No	17/7,514 (0.2)	Ref					
Yes	1/163 (0.6)	2.72	0.36–20.58	0.33			
DAA							
No	18/7,672 (0.2)	Ref					
Yes	0/5 (0.0)	–	–	–			

^*^p < 0.05

*ALT* alanine aminotransferase, *AST* aspartate aminotransferase, *BIL(T)* total bilirubin, *CI* confidence interval, *CVD* cardiovascular disease, *DAA* direct-acting agents, *eGFR* estimated glomerular filtration rate by the Modification of Diet in Renal Disease equation, *FIB-4* fibrosis-4, *HCC* hepatocellular carcinoma, *HIV* human immunodeficiency virus, *GLE/PIB* glecaprevir/pibrentasvir, *IFN* interferon, *OR* odds ratio, *PWID* persons who inject drugs, *SOF/VEL* sofosbuvir/velpatasvir

### Treatment simplification models by different eligibility criteria

The risk factors identified above for Grades 2–4 abnormal elevations in ALT/AST/BIL(T) are not included in the AASLD-IDSA or the EASL criteria, and might serve as additional indicators for separating patients with different safety management needs. As such, we applied these risk factors (except GLE/PIB usage) as a hypothetical set of criteria (“the TACR testing criteria”, defined as age > 70, presence of HCC, BIL(T) > 1.2 mg/dL, eGFR < 60 mL/min/1.73 m^2^, and FIB-4 > 3.25) back onto the study population for analyses of laboratory abnormalities and model goodness of fit. Similar analyses were also conducted using the AASLD-IDSA and the EASL criteria, to compare the three sets of criteria in predicting the occurrence of abnormal elevations in ALT/AST/BIL(T) in the current sample.

As shown in Table 5, when applying the AASLD-IDSA criteria, 6,697 patients in the TACR sample would qualify for simplified treatment (simplified-in) and 980 patients would not (simplified-out). A significantly higher odds of Grades 2–4 laboratory abnormalities in ALT/AST/BIL(T) was detected in the simplified-out group (3.9% vs 1.6%; OR = 2.46, *p* < 0.01). The corrected AIC (AICc) and BIC values for the model were 1431.25 and 1445.14, respectively. Under the EASL criteria, almost all patients would be simplified-in, precluding the analyses of the odds of laboratory abnormalities and model goodness of fit. With the TACR testing criteria, 4,172 and 3,505 patients would be simplified-in and simplified-out, respectively, with a significantly higher odds of Grades 2–4 ALT/AST/BIL(T) abnormalities in the simplified-out group (3.3% vs 0.7%; OR = 4.53, *p* < 0.01). The AICc and BIC values were 1381.18 and 1395.69, respectively. For Grades 3–4 abnormalities in ALT/AST/BIL(T), none of the three models showed meaningful separation by odds of occurrence between the simplified-in and simplified-out groups**.** Results of risk factor analyses for Grades 2–4 and Grades 3–4 laboratory abnormalities among patients who were simplified-in by TACR testing criteria can be found in Supplementary Tables 8 and 9.

**Table 5:** Comparing the three sets of criteria in the ability to predict the occurrence of abnormal elevation in ALT, AST, or BIL(T)

	Model 1 AASLD-IDSA criteria				Model 2 EASL criteria				Model 3 TACR testing criteria^a^			
	n/N (%)	OR	95% CI	P	n/N (%)	OR	95% CI	P	n/N (%)	OR	95% CI	P
Grades 2–4 laboratory abnormalities in ALT/AST/BIL(T)												
Simplified-in	108/6,697 (1.6)	Ref			146/7,639 (1.9)	Ref			31/4,172 (0.7)	Ref		
Simplified-out	38/980 (3.9)	2.46	1.69–3.58	< 0.01	0/38 (0.0)	–	–	–	115/3,505 (3.3)	4.53	3.04–6.75	< 0.01
AICc	1431.25				–				1381.18			
BIC	1445.14				–				1395.69			
AUC	0.57				–				0.67			
Grades 3–4 laboratory abnormalities in ALT/AST/BIL(T)												
Simplified-in	15/6,697 (0.2)	Ref			18/7,639 (0.2)	Ref			7/4,172 (0.2)	Ref		
Simplified-out	3/980 (0.3)	1.37	0.40–4.73	0.62	0/38 (0.0)	–	–	–	11/3,505 (0.3)	1.87	0.73–4.84	0.19
AICc	257.73				–				256.23			
BIC	271.62				–				270.12			
AUC	0.52				–				0.58			

^a^TACR’s simplified-out criteria: HCC, eGFR < 60 mL/min/1.73 m^2^, BIL(T) > 1.2 mg/dL, FIB-4 > 3.25, age > 70 years old

*AASLD-IDSA* American Association for the Study of Liver Diseases and the Infectious Diseases Society of America, *AICc* corrected Akaike information criterion, *ALT* alanine aminotransferase, *AST* aspartate aminotransferase, *BIC* Bayesian information criterion, *BIL(T)* total bilirubin, *CI* confidence interval, *EASL* European Association for the Study of the Liver, eGFR, estimated glomerular filtration rate by the Modification of Diet in Renal Disease equation, *FIB-4* fibrosis-4, *OR* odds ratio, *TACR* Taiwan Hepatitis C Registry Program

## Discussion

Data from this study showed that in CHC patients who met either AASLD-IDSA or EASL criteria for simplified treatment, SOF/VEL or GLE/PIB treatment resulted in high SVR rate and low incidence of graded laboratory abnormalities in ALT/AST/BIL(T), especially Grades 3–4 abnormalities. This is consistent with the efficacy and safety of these regimens demonstrated in previous studies [11, 12, 22, 24]. More importantly, the low incidence of abnormal elevations in liver function parameters among this patient population confirms the safety of guidelines-proposed simplified treatment algorithms under specialist setting, providing large-scale real-world evidence for implementing these algorithms [8, 9].

**Fig. 2:**
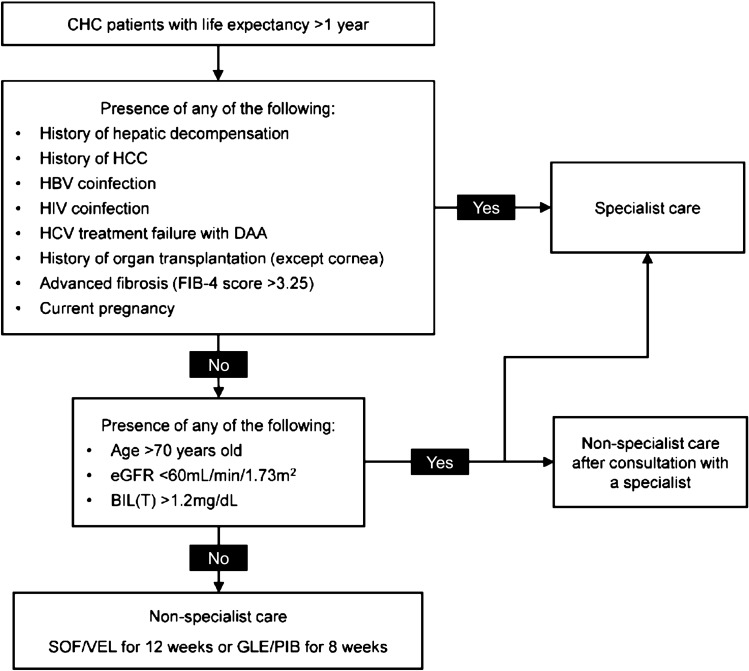
TACR algorithm for simplified HCV treatment with non-specialist care. The pan-genotypic regimens SOF/VEL (one tablet once a day with or without food for 12 weeks) and GLE/PIB (three tablets once a day with food for 8 weeks) are both recommended for simplified treatment under non-specialist care. The choice between the two regimens should be based on comprehensive consideration of the patient’s situation and need. The two regimens have different drug–drug interaction profiles, and it is important to assess potential drug–drug interactions between the DAA regimen to be used and the patient’s concomitant medications for treating comorbidities. Patient preferences and adherence should also be considered: some patients might prefer a one-tablet-daily regimen, while others might prioritize a shorter treatment duration. Cost and accessibility could also contribute to the regimen choice: the availability and cost of these regimens can vary based on the patient’s location and healthcare system, and in some regions, one regimen might be more accessible or affordable than the other. *BIL(T)* total bilirubin, *CHC* chronic hepatis C, *DAA* direct-acting agents, *eGFR* estimated glomerular filtration rate by the Modification of Diet in renal Disease equation, *FIB-4* fibrosis-4, *GLE/PIB* glecaprevir/pibrentasvir, *SOF/VEL* sofosbuvir/velpatasvir

Additionally, multivariate analyses identified patient risk factors associated with the occurrence of Grades 2–4 abnormalities in ALT/AST/BIL(T), namely age > 70 years old, presence of HCC, BIL(T) > 1.2 mg/dL, eGFR < 60 mL/min/1.73 m^2^, and FIB-4 > 3.25 (Table 3). As evidenced by the smaller AICc and BIC values of the TACR testing criteria, incorporating these factors into the treatment eligibility criteria can help better predict the occurrence of Grades 2–4 laboratory abnormalities in ALT/AST/BIL(T) in CHC patients receiving simplified pan-genotypic treatment than the AASLD-IDSA criteria (as well as the EASL criteria) (Table 5). However, if all patients with any of these risk factors are to be excluded from non-specialist care, nearly half of the patients (3,505/7,677) would require specialist care, potentially hampering the task-sharing to non-specialists. Additionally, regardless of patient characteristics, the overall incidence of graded laboratory abnormalities was low, and none of the above patient characteristics were significantly associated with higher risks of Grades 3–4 abnormalities in ALT/AST/BIL(T) (Table 4). Therefore, we adopted a holistic approach to develop a new set of criteria for simplified treatment with non-specialists for the TACR algorithm (Fig. 2) by taking into consideration (1) the AASLD-IDSA and EASL criteria, (2) the risk factors newly identified in the current analysis, as well as (3) results from previous studies or expert opinions regarding additional management needs concerning special patient populations.

The ineligibility factors common to both the AASLD-IDSA and the EASL criteria at the time of this analysis were included as ineligible factors in the TACR algorithm, namely HCC, hepatic decompensation, HBV coinfection, and current pregnancy. Patients with active HCC might respond less to DAA therapy and the priority of curative tumor therapy versus antiviral therapy needs careful evaluation by specialists [18, 25]. HCV/HBV coinfected patients are at risk of HBV reactivation during and after DAA therapy and may even develop hepatic failure if cirrhotic at baseline, thus warranting specialist care [26].

HIV coinfection was considered as an ineligibility factor by AASLD-IDSA but not EASL at the time of this analysis [8, 9]. Although it was not identified as a risk factor in this study, and AASLD-IDSA removed it from the exclusion criteria for simplified treatment in the October 2022 update [27], we still recommend patients with HIV coinfection to be managed by specialists for the following reasons: firstly, patients with HIV coinfection should also receive anti-HIV medications, which may result in complex drug-drug interactions; secondly, multiple studies have shown that HIV-coinfected CHC patients are at higher risks of HCV reinfection following successful treatment [9, 28].

Similarly, DAA treatment failure (also with inconsistent recommendations by the two existing guidelines) was added into the TACR algorithm as an ineligibility factor as a previous study demonstrated high rates of drug resistance among patients with DAA failure [29]. Patients with a history of organ transplantation (except for cornea) are ineligible for non-specialist care due to potential drug–drug interaction between DAAs and immunosuppressants [9]. Furthermore, as patients with FIB-4 > 3.25 are at increased risk of HCC and require careful HCC surveillance by specialists [30], FIB-4 > 3.25 was also incorporated as an ineligibility factor. The other risk factors identified in this study (age > 70 years old, BIL(T) > 1.2 mg/dL, and eGFR < 60 mL/min/1.73 m^2^) were incorporated as conditional ineligibility factors in the TACR algorithm: patients with these risk factors should be managed by specialists or by non-specialists only after consultation with a specialist, and with close monitoring.

Decentralization of HCV treatment delivery and task-sharing to non-specialists can increase treatment uptake, with similar high SVR rates compared with specialist care and cost-effectiveness [4, 5]. With appropriate criteria to identify patients who can receive simplified HCV treatment prescribed by non-specialists, and additional precautions for those with slightly higher safety management needs, the TACR algorithm may help promote HCV treatment uptake while minimizing potential safety concerns, generating huge health and economic benefits. Of course, other barriers to decentralization, such as the lack of interest of general practitioners and addiction specialists and unwillingness of some specialists to cede control, will also need to be addressed. To overcome these barriers, appropriate education and training of non-specialists would be crucial to raise their interest and proficiency, and also to help instill confidence in specialists regarding task-sharing to non-specialists. Additionally, efforts would be needed to improve diagnosis, such that more people living with HCV can receive appropriate treatment.

On a side note, in this study, GLE/PIB usage was associated with higher risks of abnormal elevations in liver function parameters (both Grades 2–4 and Grades 3–4) compared with SOF/VEL usage. Additionally, a similar difference was observed among patients who were simplified-in according to TACR testing criteria for the incidence of Grades 2–4 abnormalities in ALT/AST/BIL(T) (Supplementary Table 8). This difference was mainly due to elevations in BIL(T) but not in AST/ALT. Among GLE/PIB-treated patients, age > 70 was a prominent risk factor identified for Grades 2–4 abnormalities in BIL(T). Several factors identified in univariate but not multivariate analysis were comorbid conditions for which advanced age is a known risk factor, including the presence of diabetes, hypertension, cardiovascular disease, and baseline eGFR < 60 mL/min/1.73 m^2^ (Supplementary Table 7). The other risk factors identified among GLE/PIB-treated patients were the presence of HCC, FIB-4 > 3.25, and baseline BIL(T) > 1.2 mg/dL. Considering these results, the use of GLE/PIB in patients with the identified risk factors might warrant careful assessment and monitoring by specialists.

Notwithstanding the above regimen-associated differential risk profile, the incidences of abnormal elevations in ALT/AST/BIL(T) were low regardless of the pan-genotypic DAA regimen used. Among patients treated with either regimen, only one in each regimen group discontinued treatment (Supplementary Table 1). In addition, with the TACR simplified-in criteria, there was no difference in the risk of Grades 3–4 abnormalities in ALT/AST/BIL(T) between SOF/VEL and GLE/PIB usages (Supplementary Table 9). As such, both pan-genotypic regimens are recommended for TACR simplified-in patients with non-specialist care. While the two pan-genotypic regimens are similar in efficacy, they differ in treatment duration, posology, and drug–drug interaction (DDI) profiles, and the clinical decision of choosing between the two would depend on comprehensive consideration of the patient’s need (see also Fig. 2 legend). As endorsed by society guidelines, when treating HCV infection in patients with comorbidities, thorough DDI evaluation prior to DAA initiation should be conducted and the choice of DAAs should be considered for those presenting the least potential DDIs with concomitant medications and causing the least interruption to the treatment of underlying diseases. Patient perspectives should also be taken into account, including patients’ preference pertaining to treatment duration, pill burden, and/or convenience of use, so as to choose a regimen that ensures high treatment adherence and completion of DAA treatment [31, 32].

This study has limitations. First, despite a large sample size, as the study population was limited to CHC patients in Taiwan, more data may be needed to assess whether the TACR criteria can be applied to CHC patients in other countries. Second, data gathered in this study reflect HCV management under specialist care; therefore, future studies can further validate this algorithm under non-specialist setting. Such validation would also be relevant for task-shifting to specialists other than hepatologists, gastroenterologists, and infectious disease specialists. For example, patients with diabetes mellitus (DM) are at higher risk of HCV infection and represent a special population for HCV micro-elimination, and TASL has been collaborating with DM societies to promote HCV management among DM patients by DM specialists, for whom the validation of such an algorithm as presented in this manuscript would likely be of clinical relevance [33].

All in all, to our knowledge, this is the first large-scale real-world study validating the safety of implementing the simplified treatment algorithms proposed by AASLD-IDSA and EASL. Based on these real-world data, we also developed the TACR algorithm for CHC patients who are eligible for simplified treatment with non-specialist care. The algorithm can provide important guidance in the effort to decentralize HCV treatment delivery and promote task sharing to non-specialists, which would be an important step toward HCV elimination.

### Supplementary Information

Below is the link to the electronic supplementary material.

Supplementary file1 (DOCX 136 kb)

## Data Availability

The data that support the findings of this study will be made available to researchers who provide a methodologically sound proposal on request from the corresponding authors. The data are not publicly available due to privacy and ethical restrictions.
