# Supplementary material for: An algorithm for simplified hepatitis C virus treatment with non-specialist care based on nation-wide data from Taiwan
Source: Hepatol Int. 2024 Jan 21;18(2):461–75. doi: 10.1007/s12072-023-10609-7 (PMC11014878; doi:10.1007/s12072-023-10609-7)
Supplement: Supplementary file 1 — Supplementary file1 (DOCX 136 kb) [file 12072_2023_10609_MOESM1_ESM.docx]

**SUPPLEMENTARY MATERIALS**

**Article Title:** An algorithm for simplified hepatitis C virus treatment with non-specialist care based on nation-wide data from Taiwan

**Journal:** Hepatology International

**Authors:** Ming-Lung Yu, Chi‐Ming Tai, Lein-Ray Mo, Hsing-Tao Kuo, Chung-Feng Huang, Kuo-Chih Tseng, Ching-Chu Lo, Ming-Jong Bair, Szu-Jen Wang, Jee-Fu Huang, Ming-Lun Yeh, Chun-Ting Chen, Ming-Chang Tsai, Chien-Wei Huang, Pei-Lun Lee, Tzeng-Hue Yang, Yi-Hsiang Huang, Lee-Won Chong, Chien-Lin Chen, Chi-Chieh Yang, Chao-Hung Hung, Sheng‐Shun Yang, Pin-Nan Cheng, Tsai-Yuan Hsieh, Jui-Ting Hu, Wen-Chih Wu, Chien-Yu Cheng, Guei-Ying Chen, Guo-Xiong Zhou, Wei-Lun Tsai, Chien-Neng Kao, Chih-Lang Lin, Chia-Chi Wang, Ta-Ya Lin, Chih‐Lin Lin, Wei-Wen Su, Tzong-Hsi Lee, Te-Sheng Chang, Chun-Jen Liu, Chia-Yen Dai, Chi-Yi Chen, Jia-Horng Kao, Han-Chieh Lin, Wan-Long Chuang, Cheng-Yuan Peng

**Corresponding Authors:** Cheng-Yuan Peng, China Medical University Hospital, cypeng@mail.cmuh.org.tw; Wan-Long Chuang, Kaohsiung Medical University Hospital, waloch@kmu.edu.tw

# Supplementary Table 1 List of patients with Grades 3–4 laboratory abnormalities in ALT/AST/BIL(T)

| **No.** | **Sex** | **LC** | **HCC** | **HCV  GT** | **Regimen** | **Laboratory Abnormalities** | **Peak  BIL(T)** | **Peak  AST** | **Peak  ALT** | **Peak  Time** | **DAA DC** | **SVR** | **AE/SAE** | **Comorbidity** |
| --- | --- | --- | --- | --- | --- | --- | --- | --- | --- | --- | --- | --- | --- | --- |
| 1 | F | No | No | 1 | SOF/VEL | Grade 3: AST + ALT |  | 579 | 754 | Week 4 | No | Yes |  | Lung cancer; Chronic ischemic heart disease |
| 2 | M | No | No | 6 | GLE/PIB | Grade 3: AST |  | 258 |  | Week 2 | No | Unknown |  |  |
| 3 | M | No | No | 1 | GLE/PIB | Grade 3: ALT |  |  | 557 | Month 3 | No | No | Fatigue |  |
| 4 | M | No | Yes | 2 | GLE/PIB | Grade 3: BIL(T) | 3.7 |  |  | Week 2 | No | Yes | Jaundice |  |
| 5 | M | No | No | 1 | GLE/PIB | Grade 3: AST + ALT |  | 501 | 430 | Month 3 | No | Unknown |  |  |
| 6 | F | No | No | 1 | GLE/PIB | Grade 3: AST + ALT |  | 405 | 448 | Week 4 | No | Yes |  |  |
| 7 | M | No | No | 1 | GLE/PIB | Grade 3: AST + ALT |  | 1473 | 2000 | Week 4 | No | Yes |  |  |
| 8 | F | No | No | 2 | SOF/VEL | Grade 3: AST |  | 402 |  | Week 4 | No | Yes |  |  |
| 9 | M | Yes | No | 1 | SOF/VEL | Grade 3: ALT |  |  | 440 | EOT | No | Yes |  |  |
| 10 | F | No | No | 6 | SOF/VEL | Grade 3: ALT |  |  | 210 | Week 4 | No | Yes |  |  |
| 11 | M | No | No | 1 | GLE/PIB | Grade 3: BIL(T) | 3.9 |  |  | Week 4 | No | Yes | Jaundice |  |
| 12 | F | Yes | No | 1+2 | GLE/PIB | Grade 3: BIL(T) | 5.4 |  |  | Week 4 | No | Yes | Jaundice |  |
| 13 | F | No | No | 2 | SOF/VEL | Grade 3: ALT |  |  | 225 | Week 4 | No | Yes |  |  |
| 14 | M | No | No | 6 | SOF/VEL | Grade 3: AST  Grade 4: ALT |  | 633 | 1420 | Week 4 | Yes | Yes |  | Thyroid cancer |
| 15 | M | Yes | No | 2 | GLE/PIB | Grade 3: BIL(T) | 3.65 |  |  | Week 4 | No | Yes | Jaundice |  |
| 16 | M | Yes | No | 1 | SOF/VEL | Grade 3: AST |  | 842 |  | Month 3 | No | Yes |  |  |
| 17 | M | No | No | 2 | GLE/PIB | Grade 3: BIL(T) | 3.93 |  |  | Week 4 | No | Yes | Jaundice | Gastric cancer |
| 18 | M | Yes | No | 3 | GLE/PIB | Grade 3: BIL(T) | 10 |  |  | Week 4 | Yes | Unknown | Ascites, hepatic failure, death | Alcoholism |

AE, adverse event; ALT, alanine aminotransferase; AST, aspartate aminotransferase; BIL(T), total bilirubin; DAA, direct-acting agents; DC, discontinuation; EOT, end of treatment; GLE/PIB, glecaprevir/pibrentasvir; GT, genotype; HCC, hepatocellular carcinoma; LC, liver cirrhosis; SAE, severe adverse event; SOF/VEL, sofosbuvir/velpatasvir; SVR, sustained virological response

# Supplementary Table 2 Risk factors associated with Grades 2–4 laboratory abnormalities in ALT

|  | **Grades 2–4 ALT, n/N (%)** | **Univariate** | | | **Multivariate** | | |
| --- | --- | --- | --- | --- | --- | --- | --- |
|  |  | **OR** | **95% CI** | **P** | **OR** | **95% CI** | **P** |
| **Age** | |  |  |  |  |  |  |
| ≤70 | 14/5,966 (0.2) | Ref |  |  |  |  |  |
| >70 | 7/1,711 (0.4) | 1.75 | 0.70–4.33 | 0.23 |  |  |  |
| **Sex** | |  |  |  |  |  |  |
| Female | 8/3,611 (0.2) | Ref |  |  |  |  |  |
| Male | 13/4,066 (0.3) | 1.44 | 0.60–3.49 | 0.41 |  |  |  |
| **Diabetes** | |  |  |  |  |  |  |
| No | 18/6,432 (0.3) | Ref |  |  |  |  |  |
| Yes | 3/1,245 (0.2) | 0.86 | 0.25–2.92 | 0.80 |  |  |  |
| **Hypertension** | |  |  |  |  |  |  |
| No | 14/5,431 (0.3) | Ref |  |  |  |  |  |
| Yes | 7/2,246 (0.3) | 1.21 | 0.49–3.00 | 0.68 |  |  |  |
| **Hyperlipidemia** | |  |  |  |  |  |  |
| No | 18/6,826 (0.3) | Ref |  |  |  |  |  |
| Yes | 3/851 (0.4) | 1.34 | 0.39–4.55 | 0.64 |  |  |  |
| **CVD** |  |  |  |  |  |  |  |
| No | 19/7,033 (0.3) | Ref |  |  |  |  |  |
| Yes | 2/644 (0.3) | 1.15 | 0.27–4.95 | 0.85 |  |  |  |
| **PWID** |  |  |  |  |  |  |  |
| No | 19/6,859 (0.3) | Ref |  |  |  |  |  |
| Yes | 2/818 (0.2) | 0.88 | 0.21–3.79 | 0.87 |  |  |  |
| **HIV** |  |  |  |  |  |  |  |
| No | 20/7,467 (0.3) | Ref |  |  |  |  |  |
| Yes | 1/210 (0.5) | 1.78 | 0.24–13.34 | 0.58 |  |  |  |
| **HCC** |  |  |  |  |  |  |  |
| No | 19/7,394 (0.3) | Ref |  |  |  |  |  |
| Yes | 2/283 (0.7) | 2.76 | 0.64–11.91 | 0.17 |  |  |  |
| **Baseline BIL(T)** | |  |  |  |  |  |  |
| ≤1.2 | 19/6,837 (0.3) | Ref |  |  |  |  |  |
| >1.2 | 2/840 (0.2) | 0.86 | 0.20–3.68 | 0.84 |  |  |  |
| **Baseline AST** | |  |  |  |  |  |  |
| ≤200 | 14/7,523 (0.2) | Ref |  |  |  |  |  |
| >200 | 1/154 (0.7) | 2.45 | 0.33–18.39 | 0.38 |  |  |  |
| **Baseline ALT** | |  |  |  |  |  |  |
| ≤200 | 13/7,298 (0.2) | Ref |  |  |  |  |  |
| >200 | 2/379 (0.5) | 2.03 | 0.47–8.76 | 0.34 |  |  |  |
| **FIB-4** |  |  |  |  |  |  |  |
| ≤3.25 | 17/6,008 (0.3) | Ref |  |  |  |  |  |
| >3.25 | 4/1,650 (0.2) | 0.86 | 0.29–2.55 | 0.78 |  |  |  |
| **eGFR** |  |  |  |  |  |  |  |
| ≥60 | 15/6,573 (0.2) | Ref |  |  | Ref |  |  |
| <60 | 6/1,083 (0.6) | 2.44 | 0.94–6.29 | 0.07 | 2.44 | 0.94–6.29 | 0.07* |
| **Regimen** | |  |  |  |  |  |  |
| SOF/VEL | 12/5,228 (0.2) | Ref |  |  |  |  |  |
| GLE/PIB | 9/2,449 (0.4) | 1.60 | 0.67–3.81 | 0.29 |  |  |  |
| **Treatment experience** | |  |  |  |  |  |  |
| **IFN** |  |  |  |  |  |  |  |
| No | 21/7,514 (0.3) | - | - | - |  |  |  |
| Yes | 0/163 (0.0) | - | - | - |  |  |  |
| **DAA** |  |  |  |  |  |  |  |
| No | 21/7,672 (0.3) | - | - | - |  |  |  |
| Yes | 0/5 (0.0) | - | - | - |  |  |  |

*p<0.05; ALT, alanine aminotransferase; AST, aspartate aminotransferase; BIL(T), total bilirubin; CI, confidence interval; CVD, cardiovascular disease; DAA, direct acting agents; eGFR, estimated glomerular filtration rate by the Modification of Diet in Renal Disease equation; FIB-4, fibrosis-4; GLE/PIB, glecaprevir/pibrentasvir; HCC, hepatocellular carcinoma; HIV, human immunodeficiency virus; IFN, interferon; OR, odds ratio; PWID, persons who inject drugs; SOF/VEL, sofosbuvir/velpatasvir

# Supplementary Table 3 Risk factors associated with Grades 2–4 laboratory abnormalities in AST

|  | **Grades 2–4 AST, n/N (%)** | **Univariate** | | | **Multivariate** | | |
| --- | --- | --- | --- | --- | --- | --- | --- |
|  |  | **OR** | **95% CI** | **P** | **OR** | **95% CI** | **P** |
| **Age** | |  |  |  |  |  |  |
| ≤70 | 10/5,966 (0.2) | Ref |  |  |  |  |  |
| >70 | 5/1,711 (0.3) | 1.74 | 0.60–5.11 | 0.31 |  |  |  |
| **Sex** | |  |  |  |  |  |  |
| Female | 4/3,611 (0.1) | Ref |  |  |  |  |  |
| Male | 11/4,066 (0.3) | 2.44 | 0.78–7.69 | 0.13 |  |  |  |
| **Diabetes** | |  |  |  |  |  |  |
| No | 13/6,432 (0.2) | Ref |  |  |  |  |  |
| Yes | 2/1,245 (0.2) | 0.79 | 0.18–3.52 | 0.77 |  |  |  |
| **Hypertension** | |  |  |  |  |  |  |
| No | 10/5,431 (0.2) | Ref |  |  |  |  |  |
| Yes | 5/2,246 (0.2) | 1.21 | 0.41–3.54 | 0.73 |  |  |  |
| **Hyperlipidemia** | |  |  |  |  |  |  |
| No | 13/6,826 (0.2) | Ref |  |  |  |  |  |
| Yes | 2/851 (0.2) | 1.23 | 0.28–5.49 | 0.78 |  |  |  |
| **CVD** |  |  |  |  |  |  |  |
| No | 15/7,033 (0.2) | Ref |  |  |  |  |  |
| Yes | 0/644 (0.0) | - | - | - |  |  |  |
| **PWID** |  |  |  |  |  |  |  |
| No | 12/6,859 (0.2) | Ref |  |  |  |  |  |
| Yes | 3/818 (0.4) | 2.10 | 0.59–7.46 | 0.26 |  |  |  |
| **HIV** |  |  |  |  |  |  |  |
| No | 14/7,467 (0.2) | Ref |  |  |  |  |  |
| Yes | 1/210 (0.5) | 2.55 | 0.33–19.46 | 0.37 |  |  |  |
| **HCC** |  |  |  |  |  |  |  |
| No | 14/7,394 (0.2) | Ref |  |  |  |  |  |
| Yes | 1/283 (0.4) | 1.87 | 0.24–14.26 | 0.55 |  |  |  |
| **Baseline BIL(T)** | |  |  |  |  |  |  |
| ≤1.2 | 12/6,837 (0.2) | Ref |  |  |  |  |  |
| >1.2 | 3/840 (0.4) | 2.04 | 0.57–7.24 | 0.27 |  |  |  |
| **Baseline AST** | |  |  |  |  |  |  |
| ≤200 | 14/7,523 (0.2) | Ref |  |  |  |  |  |
| >200 | 1/154 (0.7) | 3.51 | 0.46–26.83 | 0.23 |  |  |  |
| **Baseline ALT** | |  |  |  |  |  |  |
| ≤200 | 13/7,298 (0.2) | Ref |  |  |  |  |  |
| >200 | 2/379 (0.5) | 2.97 | 0.67–13.22 | 0.15 |  |  |  |
| **FIB-4** |  |  |  |  |  |  |  |
| ≤3.25 | 10/6,008 (0.2) | Ref |  |  |  |  |  |
| >3.25 | 5/1,650 (0.3) | 1.82 | 0.62–5.34 | 0.27 |  |  |  |
| **eGFR** |  |  |  |  |  |  |  |
| ≥60 | 12/6,573 (0.2) | Ref |  |  |  |  |  |
| <60 | 3/1,083 (0.2) | 1.52 | 0.43–5.39 | 0.52 |  |  |  |
| **Regimen** | |  |  |  |  |  |  |
| SOF/VEL | 8/5,228 (0.2) | Ref |  |  |  |  |  |
| GLE/PIB | 7/2,449 (0.3) | 1.87 | 0.68–5.16 | 0.23 |  |  |  |
| **Treatment experience** | |  |  |  |  |  |  |
| **IFN** |  |  |  |  |  |  |  |
| No | 15/7,514 (0.2) | - | - | - |  |  |  |
| Yes | 0/163 (0.0) | - | - | - |  |  |  |
| **DAA** |  |  |  |  |  |  |  |
| No | 15/7,672 (0.2) | - | - | - |  |  |  |
| Yes | 0/5 (0.0) | - | - | - |  |  |  |

ALT, alanine aminotransferase; AST, aspartate aminotransferase; BIL(T), total bilirubin; CI, confidence interval; CVD, cardiovascular disease; DAA, direct acting agents; eGFR, estimated glomerular filtration rate by the Modification of Diet in Renal Disease equation; FIB-4, fibrosis-4; GLE/PIB, glecaprevir/pibrentasvir; HCC, hepatocellular carcinoma; HIV, human immunodeficiency virus; IFN, interferon; OR, odds ratio; PWID, persons who inject drugs; SOF/VEL, sofosbuvir/velpatasvir

# Supplementary Table 4 Risk factors associated with Grades 2–4 laboratory abnormalities in BIL(T)

|  | **Grades 2–4 BIL(T), n/N (%)** | **Univariate** | | | **Multivariate** | | |
| --- | --- | --- | --- | --- | --- | --- | --- |
|  |  | **OR** | **95% CI** | **P** | **OR** | **95% CI** | **P** |
| **Age** | |  |  |  |  |  |  |
| ≤70 | 73/5,966 (1.2) | Ref |  |  | Ref |  |  |
| >70 | 49/1,711 (2.9) | 2.38 | 1.65–3.43 | <0.01* | 1.62 | 1.05–2.49 | 0.03* |
| **Sex** | |  |  |  |  |  |  |
| Female | 54/3,611 (1.5) | Ref |  |  |  |  |  |
| Male | 68/4,066 (1.7) | 1.12 | 0.62–1.28 | 0.54 |  |  |  |
| **Diabetes** | |  |  |  |  |  |  |
| No | 97/6,432 (1.5) | Ref |  |  |  |  |  |
| Yes | 25/1,245 (2.0) | 1.34 | 0.86–2.09 | 0.20 |  |  |  |
| **Hypertension** | |  |  |  |  |  |  |
| No | 66/5,431 (1.2) | Ref |  |  | Ref |  |  |
| Yes | 56/2,246 (2.5) | 2.08 | 1.45–2.98 | <0.01* | 1.37 | 0.90–2.07 | 0.14 |
| **Hyperlipidemia** | |  |  |  |  |  |  |
| No | 106/6,826 (1.6) | Ref |  |  |  |  |  |
| Yes | 16/851 (1.9) | 1.21 | 0.71–2.07 | 0.47 |  |  |  |
| **CVD** |  |  |  |  |  |  |  |
| No | 101/7,033 (1.4) | Ref |  |  | Ref |  |  |
| Yes | 21/644 (3.3) | 2.31 | 1.44–3.73 | <0.01* | 1.47 | 0.85–2.53 | 0.17 |
| **PWID** |  |  |  |  |  |  |  |
| No | 116/6,859 (1.7) | Ref |  |  | Ref |  |  |
| Yes | 6/818 (0.7) | 0.43 | 0.19–0.98 | 0.04* | 0.74 | 0.32–1.73 | 0.49 |
| **HIV** |  |  |  |  |  |  |  |
| No | 119/7,467 (1.6) | Ref |  |  |  |  |  |
| Yes | 3/210 (1.4) | 0.89 | 0.28–2.84 | 0.85 |  |  |  |
| **HCC** |  |  |  |  |  |  |  |
| No | 104/7,394 (1.4) | Ref |  |  | Ref |  |  |
| Yes | 18/283 (6.4) | 4.76 | 2.84–7.97 | <0.01* | 2.74 | 1.55–4.85 | <0.01* |
| **Baseline BIL(T)** | |  |  |  |  |  |  |
| ≤1.2 | 75/6,837 (1.1) | Ref |  |  | Ref |  |  |
| >1.2 | 47/840 (5.6) | 5.34 | 3.68–7.75 | <0.01* | 5.65 | 3.76–8.48 | <0.01* |
| **Baseline AST** | |  |  |  |  |  |  |
| ≤200 | 118/7,523 (1.6) | Ref |  |  |  |  |  |
| >200 | 4/154 (2.6) | 1.67 | 0.61–4.59 | 0.32 |  |  |  |
| **Baseline ALT** | |  |  |  |  |  |  |
| ≤200 | 117/7,298 (1.6) | Ref |  |  |  |  |  |
| >200 | 5/379 (1.3) | 0.82 | 0.33–2.02 | 0.67 |  |  |  |
| **FIB-4** |  |  |  |  |  |  |  |
| ≤3.25 | 64/6,008 (1.1) | Ref |  |  | Ref |  |  |
| >3.25 | 58/1,650 (3.5) | 3.38 | 2.36–4.85 | <0.01* | 2.09 | 1.39–3.14 | <0.01* |
| **eGFR** |  |  |  |  |  |  |  |
| ≥60 | 88/6,573 (1.3) | Ref |  |  | Ref |  |  |
| <60 | 34/1,083 (3.1) | 2.39 | 1.60–3.57 | <0.01* | 1.54 | 0.98–2.41 | 0.06 |
| **Regimen** | |  |  |  |  |  |  |
| SOF/VEL | 40/5,228 (0.8) | Ref |  |  | Ref |  |  |
| GLE/PIB | 82/2,449 (3.4) | 4.49 | 3.07–6.58 | <0.01* | 6.02 | 4.02–9.01 | <0.01* |
| **Treatment experience** | |  |  |  |  |  |  |
| **IFN** |  |  |  |  |  |  |  |
| No | 120/7,514 (1.6) | Ref |  |  |  |  |  |
| Yes | 2/163 (1.2) | 0.77 | 0.19–3.12 | 0.71 |  |  |  |
| **DAA** |  |  |  |  |  |  |  |
| No | 121/7,672 (1.6) | Ref |  |  | Ref |  |  |
| Yes | 1/5 (20.0) | 15.60 | 1.73–140.61 | 0.01* | 8.38 | 0.85–84.40 | 0.07 |

*p<0.05; ALT, alanine aminotransferase; AST, aspartate aminotransferase; BIL(T), total bilirubin; CI, confidence interval; CVD, cardiovascular disease; DAA, direct acting agents; eGFR, estimated glomerular filtration rate by the Modification of Diet in Renal Disease equation; FIB-4, fibrosis-4; GLE/PIB, glecaprevir/pibrentasvir; HCC, hepatocellular carcinoma; HIV, human immunodeficiency virus; IFN, interferon; OR, odds ratio; PWID, persons who inject drugs; SOF/VEL, sofosbuvir/velpatasvir

# Supplementary Table 5 Risk factors associated with Grades 2–4 laboratory abnormalities in ALT/AST/BIL(T) among patients treated with SOF/VEL (N=5,528)

|  | **Grades 2–4 ALT/AST/BIL(T), n/N (%)** | **Univariate** | | | **Multivariate** | | |
| --- | --- | --- | --- | --- | --- | --- | --- |
|  |  | **OR** | **95% CI** | **P** | **OR** | **95% CI** | **P** |
| **Age** | |  |  |  |  |  |  |
| ≤70 | 32/3,966 (0.8) | Ref |  |  | Ref |  |  |
| >70 | 22/1,262 (1.7) | 2.18 | 1.26–3.77 | <0.01* | 1.29 | 0.70–2.38 | 0.41 |
| **Sex** | |  |  |  |  |  |  |
| Female | 20/2,560 (0.8) | Ref |  |  |  |  |  |
| Male | 34/2,668 (1.3) | 1.64 | 0.94–2.86 | 0.08 |  |  |  |
| **Diabetes** | |  |  |  |  |  |  |
| No | 47/4,380 (1.1) | Ref |  |  |  |  |  |
| Yes | 7/848 (0.8) | 0.77 | 0.34–1.70 | 0.52 |  |  |  |
| **Hypertension** | |  |  |  |  |  |  |
| No | 35/3,696 (1.0) | Ref |  |  |  |  |  |
| Yes | 19/1,532 (1.2) | 1.31 | 0.75–2.30 | 0.34 |  |  |  |
| **Hyperlipidemia** | |  |  |  |  |  |  |
| No | 49/4,622 (1.1) | Ref |  |  |  |  |  |
| Yes | 5/606 (0.8) | 0.78 | 0.31–1.96 | 0.59 |  |  |  |
| **CVD** |  |  |  |  |  |  |  |
| No | 48/4,757 (1.0) | Ref |  |  |  |  |  |
| Yes | 6/471 (1.3) | 1.27 | 0.54–2.97 | 0.59 |  |  |  |
| **PWID** |  |  |  |  |  |  |  |
| No | 51/4,660 (1.1) | Ref |  |  |  |  |  |
| Yes | 3/568 (0.5) | 0.48 | 0.15–1.54 | 0.22 |  |  |  |
| **HIV** |  |  |  |  |  |  |  |
| No | 52/5,072 (1.0) | Ref |  |  |  |  |  |
| Yes | 2/156 (1.3) | 1.24 | 0.30–5.19 | 0.76 |  |  |  |
| **HCC** |  |  |  |  |  |  |  |
| No | 43/5,012 (0.9) | Ref |  |  | Ref |  |  |
| Yes | 11/216 (5.1) | 6.20 | 3.15–12.20 | <0.01* | 2.76 | 1.30–5.84 | <0.01* |
| **Baseline BIL(T)** | |  |  |  |  |  |  |
| ≤1.2 | 26/4,604 (0.6) | Ref |  |  |  |  |  |
| >1.2 | 28/624 (4.5) | 8.27 | 4.81–14.20 | <0.01* | 6.49 | 3.66–11.53 | <0.01* |
| **Baseline AST** | |  |  |  |  |  |  |
| ≤200 | 52/5,101 (1.0) | Ref |  |  |  |  |  |
| >200 | 2/127 (1.6) | 1.55 | 0.37–6.45 | 0.54 |  |  |  |
| **Baseline ALT** | |  |  |  |  |  |  |
| ≤200 | 52/4,944 (1.1) | Ref |  |  |  |  |  |
| >200 | 2/284 (0.7) | 0.67 | 0.16–2.75 | 0.58 |  |  |  |
| **FIB-4** |  |  |  |  |  |  |  |
| ≤3.25 | 23/3,994 (0.6) | Ref |  |  | Ref |  |  |
| >3.25 | 31/1,219 (2.5) | 4.50 | 2.62–7.76 | <0.01* | 2.19 | 1.19–4.03 | 0.01* |
| **eGFR** |  |  |  |  |  |  |  |
| ≥60 | 41/4,561 (0.9) | Ref |  |  | Ref |  |  |
| <60 | 13/654 (2.0) | 2.24 | 1.19–4.20 | <0.01* | 1.93 | 0.98–3.79 | 0.06 |
| **Treatment experience** | |  |  |  |  |  |  |
| **IFN** |  |  |  |  |  |  |  |
| No | 54/5,098 (1.1) | Ref |  |  |  |  |  |
| Yes | 0/130 (0.0) | - | - | - |  |  |  |
| **DAA** |  |  |  |  |  |  |  |
| No | 53/5,227 (1.0) | Ref |  |  |  |  |  |
| Yes | 1/1 (100.0) | - | - | - |  |  |  |

*p<0.05; ALT, alanine aminotransferase; AST, aspartate aminotransferase; BIL(T), total bilirubin; CI, confidence interval; CVD, cardiovascular disease; DAA, direct acting agents; eGFR, estimated glomerular filtration rate by the Modification of Diet in Renal Disease equation; FIB-4, fibrosis-4; HCC, hepatocellular carcinoma; HIV, human immunodeficiency virus; IFN, interferon; OR, odds ratio; PWID, persons who inject drugs; SOF/VEL, sofosbuvir/velpatasvir

# Supplementary Table 6 Risk factors associated with Grades 2–4 laboratory abnormalities in ALT/AST/BIL(T) among patients treated with GLE/PIB (n=2,449)

|  | **Grades 2–4 ALT/AST/BIL(T), n/N (%)** | **Univariate** | | | **Multivariate** | | |
| --- | --- | --- | --- | --- | --- | --- | --- |
|  |  | **OR** | **95% CI** | **P** | **OR** | **95% CI** | **P** |
| **Age** | |  |  |  |  |  |  |
| ≤70 | 57/2,000 (2.9) | Ref |  |  | Ref |  |  |
| >70 | 35/449 (7.8) | 2.88 | 1.87–4.45 | <0.01* | 1.89 | 1.15–3.12 | 0.01* |
| **Sex** | |  |  |  |  |  |  |
| Female | 42/1,051 (4.0) | Ref |  |  |  |  |  |
| Male | 50/1,398 (3.6) | 0.89 | 0.59–1.35 | 0.59 |  |  |  |
| **Diabetes** | |  |  |  |  |  |  |
| No | 69/2,052 (3.4) | Ref |  |  | Ref |  |  |
| Yes | 23/397 (5.8) | 1.77 | 1.09–2.87 | 0.02* | 1.10 | 0.62–1.94 | 0.74 |
| **Hypertension** | |  |  |  |  |  |  |
| No | 47/1,735 (2.7) | Ref |  |  | Ref |  |  |
| Yes | 45/714 (6.3) | 2.42 | 1.59–3.67 | <0.01* | 1.43 | 0.86–2.39 | 0.17 |
| **Hyperlipidemia** | |  |  |  |  |  |  |
| No | 78/2,204 (3.5) | Ref |  |  | Ref |  |  |
| Yes | 14/245 (5.7) | 1.65 | 0.92–2.96 | 0.09 | 1.12 | 0.58–2.14 | 0.74 |
| **CVD** |  |  |  |  |  |  |  |
| No | 75/2,276 (3.3) | Ref |  |  | Ref |  |  |
| Yes | 17/173 (9.8) | 3.20 | 1.84–5.55 | <0.01* | 1.73 | 0.93–3.24 | 0.09 |
| **PWID** |  |  |  |  |  |  |  |
| No | 85/2,199 (3.9) | Ref |  |  |  |  |  |
| Yes | 7/250 (2.8) | 0.72 | 0.33–1.57 | 0.40 |  |  |  |
| **HIV** |  |  |  |  |  |  |  |
| No | 89/2,395 (3.7) | Ref |  |  |  |  |  |
| Yes | 3/54 (5.6) | 1.52 | 0.47–4.98 | 0.49 |  |  |  |
| **HCC** |  |  |  |  |  |  |  |
| No | 83/2,382 (3.5) | Ref |  |  | Ref |  |  |
| Yes | 9/67 (13.4) | 4.30 | 2.06–8.97 | <0.01* | 2.38 | 1.08–5.25 | 0.03* |
| **Baseline BIL(T)** | |  |  |  |  |  |  |
| ≤1.2 | 69/2,233 (3.1) | Ref |  |  | Ref |  |  |
| >1.2 | 23/216 (10.7) | 3.74 | 2.28–6.13 | <0.01* | 3.64 | 2.15–6.17 | <0.01* |
| **Baseline AST** | |  |  |  |  |  |  |
| ≤200 | 89/2,422 (3.7) | Ref |  |  |  |  |  |
| >200 | 3/27 (11.1) | 3.28 | 0.97–11.08 | 0.06 |  |  |  |
| **Baseline ALT** | |  |  |  |  |  |  |
| ≤200 | 87/2,354 (3.7) | Ref |  |  |  |  |  |
| >200 | 5/95 (5.3) | 1.45 | 0.57–3.65 | 0.43 |  |  |  |
| **FIB-4** |  |  |  |  |  |  |  |
| ≤3.25 | 59/2,014 (2.9) | Ref |  |  | Ref |  |  |
| >3.25 | 33/431 (7.7) | 2.75 | 1.77–4.26 | <0.01* | 1.57 | 0.97–2.57 | 0.07 |
| **eGFR** |  |  |  |  |  |  |  |
| ≥60 | 65/2,012 (3.2) | Ref |  |  | Ref |  |  |
| <60 | 27/429 (6.3) | 2.01 | 1.27–3.19 | <0.01* | 1.30 | 0.76–2.22 | 0.33 |
| **Treatment experience** | |  |  |  |  |  |  |
| **IFN** |  |  |  |  |  |  |  |
| No | 90/2,416 (3.7) | Ref |  |  |  |  |  |
| Yes | 2/33 (6.1) | 1.67 | 0.39–7.08 | 0.49 |  |  |  |
| **DAA** |  |  |  |  |  |  |  |
| No | 92/2,445 (3.8) | Ref |  |  |  |  |  |
| Yes | 0/4 (0.0) | - | - | - |  |  |  |

*p<0.05; ALT, alanine aminotransferase; AST, aspartate aminotransferase; BIL(T), total bilirubin; CI, confidence interval; CVD, cardiovascular disease; DAA, direct acting agents; eGFR, estimated glomerular filtration rate by the Modification of Diet in Renal Disease equation; FIB-4, fibrosis-4; GLE/PIB, glecaprevir/pibrentasvir; HCC, hepatocellular carcinoma; HIV, human immunodeficiency virus; IFN, interferon; OR, odds ratio; PWID, persons who inject drugs

# Supplementary Table 7 Risk factors associated with Grades 2–4 laboratory abnormalities in BIL(T) among patients treated with GLE/PIB (n=2,449)

|  | **Grades 2–4 BIL(T), n/N (%)** | **Univariate** | | | **Multivariate** | | |
| --- | --- | --- | --- | --- | --- | --- | --- |
|  |  | **OR** | **95% CI** | **P** | **OR** | **95% CI** | **P** |
| **Age** | |  |  |  |  |  |  |
| ≤70 | 49/2,000 (2.5) | Ref |  |  | Ref |  |  |
| >70 | 33/449 (7.4) | 3.16 | 2.01–4.97 | <0.01* | 1.91 | 1.13–3.23 | 0.02* |
| **Sex** | |  |  |  |  |  |  |
| Female | 40/1,051 (3.8) | Ref |  |  |  |  |  |
| Male | 42/1,398 (3.0) | 0.78 | 0.82–1.98 | 0.28 |  |  |  |
| **Diabetes** | |  |  |  |  |  |  |
| No | 61/2,052 (3.0) | Ref |  |  | Ref |  |  |
| Yes | 21/397 (5.3) | 1.82 | 1.10–3.03 | 0.02* | 1.06 | 0.58–1.92 | 0.85 |
| **Hypertension** | |  |  |  |  |  |  |
| No | 39/1,735 (2.3) | Ref |  |  | Ref |  |  |
| Yes | 43/714 (6.0) | 2.79 | 1.79–4.34 | <0.01* | 1.60 | 0.93–2.75 | 0.09 |
| **Hyperlipidemia** | |  |  |  |  |  |  |
| No | 69/2,204 (3.1) | Ref |  |  | Ref |  |  |
| Yes | 13/245 (5.3) | 1.73 | 0.94–3.18 | 0.08* | 1.13 | 0.57–2.22 | 0.73 |
| **CVD** |  |  |  |  |  |  |  |
| No | 65/2,276 (2.9) | Ref |  |  | Ref |  |  |
| Yes | 17/173 (9.8) | 3.71 | 2.12–6.48 | <0.01* | 1.87 | 0.99–3.55 | 0.06 |
| **PWID** |  |  |  |  |  |  |  |
| No | 78/2,199 (3.6) | Ref |  |  |  |  |  |
| Yes | 4/250 (1.6) | 0.44 | 0.16–1.22 | 0.11 |  |  |  |
| **HIV** |  |  |  |  |  |  |  |
| No | 80/2,395 (3.3) | Ref |  |  |  |  |  |
| Yes | 2/54 (3.7) | 1.11 | 0.27–4.65 | 0.88 |  |  |  |
| **HCC** |  |  |  |  |  |  |  |
| No | 73/2,382 (3.1) | Ref |  |  | Ref |  |  |
| Yes | 9/67 (13.4) | 4.91 | 2.34–10.29 | <0.01* | 2.51 | 1.12–5.61 | 0.03* |
| **Baseline BIL(T)** | |  |  |  |  |  |  |
| ≤1.2 | 60/2,233 (2.7) | Ref |  |  | Ref |  |  |
| >1.2 | 22/216 (10.2) | 4.11 | 2.47–6.84 | <0.01* | 3.90 | 2.25–6.75 | <0.01* |
| **Baseline AST** | |  |  |  |  |  |  |
| ≤200 | 80/2,422 (3.3) | Ref |  |  |  |  |  |
| >200 | 2/27 (7.4) | 2.34 | 0.55–10.06 | 0.25 |  |  |  |
| **Baseline ALT** | |  |  |  |  |  |  |
| ≤200 | 79/2,354 (3.4) | Ref |  |  |  |  |  |
| >200 | 3/95 (3.2) | 0.94 | 0.29–3.03 | 0.92 |  |  |  |
| **FIB-4** |  |  |  |  |  |  |  |
| ≤3.25 | 49/2,014 (2.4) | Ref |  |  | Ref |  |  |
| >3.25 | 33/431 (7.7) | 3.33 | 2.11–5.24 | <0.01* | 1.85 | 1.12–3.07 | 0.02* |
| **eGFR** |  |  |  |  |  |  |  |
| ≥60 | 57/2,012 (2.8) | Ref |  |  | Ref |  |  |
| <60 | 25/429 (5.8) | 2.12 | 1.31–3.44 | <0.01* | 1.29 | 0.74–2.26 | 0.37 |
| **Treatment experience** | |  |  |  |  |  |  |
| **IFN** |  |  |  |  |  |  |  |
| No | 80/2,416 (3.3) | Ref |  |  |  |  |  |
| Yes | 2/33 (6.1) | 1.88 | 0.44–8.01 | 0.39 |  |  |  |
| **DAA** |  |  |  |  |  |  |  |
| No | 82/2,445 (3.4) | Ref |  |  |  |  |  |
| Yes | 0/4(0.0) | - |  | - |  |  |  |

*p<0.05; ALT, alanine aminotransferase; AST, aspartate aminotransferase; BIL(T), total bilirubin; CI, confidence interval; CVD, cardiovascular disease; DAA, direct acting agents; eGFR, estimated glomerular filtration rate by the Modification of Diet in Renal Disease equation; FIB-4, fibrosis-4; GLE/PIB, glecaprevir/pibrentasvir; HCC, hepatocellular carcinoma; HIV, human immunodeficiency virus; IFN, interferon; OR, odds ratio; PWID, persons who inject drugs

# Supplementary Table 8 Risk factors associated with Grades 2–4 laboratory abnormalities in ALT/AST/BIL(T) among TACR simplified-in patients (N=4,172)

|  | **Grades 2–4 ALT/AST/BIL(T), n/N (%)** | **Univariate** | | | **Multivariate** | | |
| --- | --- | --- | --- | --- | --- | --- | --- |
|  |  | **OR** | **95% CI** | **P** | **OR** | **95% CI** | **P** |
| **Sex** | |  |  |  |  |  |  |
| Female | 12/1,796 (0.7) | Ref |  |  |  |  |  |
| Male | 19/2,376 (0.8) | 1.20 | 0.58–2.48 | 0.62 |  |  |  |
| **Diabetes** | |  |  |  |  |  |  |
| No | 27/3,728 (0.7) | Ref |  |  |  |  |  |
| Yes | 4/444 (0.9) | 1.25 | 0.43–3.58 | 0.68 |  |  |  |
| **Hypertension** | |  |  |  |  |  |  |
| No | 22/3,395 (0.7) | Ref |  |  |  |  |  |
| Yes | 9/777 (1.2) | 1.80 | 0.82–3.92 | 0.14 |  |  |  |
| **Hyperlipidemia** | |  |  |  |  |  |  |
| No | 28/3,809 (0.7) | Ref |  |  |  |  |  |
| Yes | 3/363 (0.8) | 1.13 | 0.34–3.72 | 0.85 |  |  |  |
| **CVD** |  |  |  |  |  |  |  |
| No | 29/3,988 (0.7) | Ref |  |  |  |  |  |
| Yes | 2/184 (1.1) | 1.50 | 0.36–6.34 | 0.58 |  |  |  |
| **PWID** |  |  |  |  |  |  |  |
| No | 26/3,530 (0.7) | Ref |  |  |  |  |  |
| Yes | 5/642 (0.8) | 1.06 | 0.40–2.77 | 0.91 |  |  |  |
| **HIV** |  |  |  |  |  |  |  |
| No | 29/4,015 (0.7) | Ref |  |  |  |  |  |
| Yes | 2/157 (1.3) | 1.77 | 0.42–7.50 | 0.44 |  |  |  |
| **Baseline AST** | |  |  |  |  |  |  |
| ≤200 | 30/4,149 (0.7) | Ref |  |  |  |  |  |
| >200 | 1/23 (4.4) | 6.24 | 0.81–47.80 | 0.08 |  |  |  |
| **Baseline ALT** | |  |  |  |  |  |  |
| ≤200 | 28/4,019 (0.7) | Ref |  |  |  |  |  |
| >200 | 3/153 (2.0) | 2.85 | 0.86–9.48 | 0.09 |  |  |  |
| **Regimen** | |  |  |  |  |  |  |
| SOF/VEL | 7/2,752 (0.3) | Ref |  |  | Ref |  |  |
| GLE/PIB | 24/1,420 (1.7) | 6.74 | 2.90–15.68 | <0.01* | 6.74 | 2.90–15.68 | <0.01* |
| **Treatment experience** | |  |  |  |  |  |  |
| **IFN** |  |  |  |  |  |  |  |
| No | 31/4,091 (0.8) | Ref |  |  |  |  |  |
| Yes | 0/81 (0.0) | - | - | - |  |  |  |
| **DAA** |  |  |  |  |  |  |  |
| No | 31/4,170 (0.7) | Ref |  |  |  |  |  |
| Yes | 0/2 (0.0) | - | - | - |  |  |  |

*p<0.05; ALT, alanine aminotransferase; AST, aspartate aminotransferase; BIL(T), total bilirubin; CI, confidence interval; CVD, cardiovascular disease; DAA, direct acting agents; GLE/PIB, glecaprevir/pibrentasvir; HIV, human immunodeficiency virus; IFN, interferon; OR, odds ratio; PWID, persons who inject drugs; SOF/VEL, sofosbuvir/velpatasvir

# Supplementary Table 9 Risk factors associated with Grades 3–4 laboratory abnormalities in ALT/AST/BIL(T) among TACR simplified-in patients (N=4,172)

|  | **Grades 3–4 ALT/AST/BIL(T), n/N (%)** | **Univariate** | | | **Multivariate** | | |
| --- | --- | --- | --- | --- | --- | --- | --- |
|  |  | **OR** | **95% CI** | **P** | **OR** | **95% CI** | **P** |
| **Sex** | |  |  |  |  |  |  |
| Female | 3/1,796 (0.2) | Ref |  |  |  |  |  |
| Male | 4/2,376 (0.2) | 1.01 | 0.23–4.51 | 0.99 |  |  |  |
| **Diabetes** | |  |  |  |  |  |  |
| No | 7/3,728 (0.2) | Ref |  |  |  |  |  |
| Yes | 0/444 (0.0) | - | - | - |  |  |  |
| **Hypertension** | |  |  |  |  |  |  |
| No | 6/3,395 (0.2) | Ref |  |  |  |  |  |
| Yes | 1/777 (0.1) | 0.73 | 0.09–6.05 | 0.77 |  |  |  |
| **Hyperlipidemia** | |  |  |  |  |  |  |
| No | 7/3,809 (0.2) | Ref |  |  |  |  |  |
| Yes | 0/363 (0.0) | - | - | - |  |  |  |
| **CVD** |  |  |  |  |  |  |  |
| No | 7/3,988 (0.2) | Ref |  |  |  |  |  |
| Yes | 0/184 (0.0) | - | - | - |  |  |  |
| **PWID** |  |  |  |  |  |  |  |
| No | 5/3,530 (0.1) | Ref |  |  |  |  |  |
| Yes | 2/642 (0.3) | 2.20 | 0.43–11.38 | 0.35 |  |  |  |
| **HIV** |  |  |  |  |  |  |  |
| No | 7/4,015 (0.2) | Ref |  |  |  |  |  |
| Yes | 0/157 (0.0) | - | - | - |  |  |  |
| **Baseline AST** | |  |  |  |  |  |  |
| ≤200 | 6/4,149 (0.1) | Ref |  |  | Ref |  |  |
| >200 | 1/23 (4.4) | 31.39 | 3.63–271.63 | <0.01* | 31.39 | 3.63–271.63 | <0.01* |
| **Baseline ALT** | |  |  |  |  |  |  |
| ≤200 | 6/4,019 (0.2) | Ref |  |  |  |  |  |
| >200 | 1/153 (0.7) | 4.40 | 0.53–36.78 | 0.17 |  |  |  |
| **Regimen** | |  |  |  |  |  |  |
| SOF/VEL | 4/2,752 (0.1) | Ref |  |  |  |  |  |
| GLE/PIB | 3/1,420 (0.2) | 1.45 | 0.33–6.51 | 0.62 |  |  |  |
| **Treatment experience** | |  |  |  |  |  |  |
| **IFN** |  |  |  |  |  |  |  |
| No | 7/4,091 (0.2) | Ref |  |  |  |  |  |
| Yes | 0/81 (0.0) | - | - | - |  |  |  |
| **DAA** |  |  |  |  |  |  |  |
| No | 7/4,170 (0.2) | Ref |  |  |  |  |  |
| Yes | 0/2 (0.0) | - | - | - |  |  |  |

*p<0.05; ALT, alanine aminotransferase; AST, aspartate aminotransferase; BIL(T), total bilirubin; CI, confidence interval; CVD, cardiovascular disease; DAA, direct acting agents; GLE/PIB, glecaprevir/pibrentasvir; HIV, human immunodeficiency virus; IFN, interferon; OR, odds ratio; PWID, persons who inject drugs; SOF/VEL, sofosbuvir/velpatasvir
